# Comparison of implantation sites for the development of peritoneal metastasis in a colorectal cancer mouse model using non-invasive bioluminescence imaging

**DOI:** 10.1371/journal.pone.0220360

**Published:** 2019-07-31

**Authors:** Abdelkader Taibi, Jeremie Albouys, Jeremie Jacques, Marie-Laure Perrin, Catherine Yardin, Sylvaine Durand Fontanier, Sylvia M. Bardet

**Affiliations:** 1 Visceral Surgery Department, Dupuytren University Hospital, Limoges, France; 2 University Limoges, CNRS, XLIM, UMR 7252, Limoges, France; 3 Gastroenterology Department, Dupuytren University Hospital, Limoges, France; 4 Cytology and Histology Department, Dupuytren University Hospital, Limoges, France; German Cancer Research Center (DKFZ), GERMANY

## Abstract

The development of cancer mouse models is still needed for the identification and preclinical validation of novel therapeutic targets in colorectal cancer, which is the third leading cause of cancer-related deaths in Europe. The purpose of this study was to determine the most accurate tumour cell injection method to obtain suitable peritoneal metastasis (PM) for subsequent therapeutic treatments. Here, we grafted murine colon carcinoma CT-26 cells expressing luciferase into immunocompetent BALB-c mice by intravenous injection (IV group), subcutaneous injection (SC group), intraperitoneal injection after peritoneal scratching (A group) or intraperitoneal injection alone (IP group). Tumour growth was monitored by bioluminescence during the first 15 days post-grafting. The peritoneal carcinomatosis index was evaluated macroscopically, histology, immunohistochemistry and multiphoton microscopy were performed in peritoneal tumour tissue. Upon implantation, no tumour growth was observed in the IV group, similar to the non-injected group. Both the IP and SC groups showed intermediate growth rates, but the SC group produced only a single subcutaneous nodule. The A group exhibited the highest tumour growth at 15 days post-surgery. Anatomic and histologic analyses corroborated the existence of various tumour nodules, and multiphoton microscopy was used to evaluate tumour fibrosis-infiltrating cells in a non-pathologic peritoneum. In conclusion, limited PM was obtained by IP injection, whereas IP injection after peritoneal scratching led to an extensive PM murine model for evaluating new therapeutics.

## Introduction

Peritoneal metastasis (PM) (previously called peritoneal carcinomatosis) develops in 8.5–25% of patients with colorectal cancer (CRC) [1], and the peritoneum is the second most frequent site for metastasis after the liver [2, 3]. PM from CRC (PMc) is associated with high morbidity and mortality, as the majority of patients present with extensive PM (peritoneal carcinomatosis index (PCI) > 20) [4, 5]. The PCI is a classical quantitative method used by surgeons to assess the extent of peritoneal cancer throughout the peritoneal cavity, and is closely correlated with the prognosis of the patient. These patients may receive many treatments, such as modern chemotherapy with targeted therapy, cytoreduction procedures with hyperthermic intraperitoneal chemotherapy and adjuvant chemotherapy [4, 6, 7], but there is no curative treatment. Thus, many researchers have aimed to develop new multimodal therapeutic devices to treat patients with extensive PMc [8, 9].

Animal models play an important role in the study of PM development and progression and in testing innovative treatments [10]. Many mouse models of PM have been described in the literature and have led to important advancements in medical care [11, 12]. Nevertheless, no study has compared the different murine models available in the context of medical practice. Therefore, it is important to classify these animal models of limited or extensive PMc. In fact, mouse models with limited PM are particularly interesting and can be used to test new treatments before the disease aggravates symptoms that are incompatible with animal ethics. This extensive model is relevant for assessing new palliative treatments for cases of CRC progression. The aim of this study was to evaluate the implantation rate of CT-26, a type of murine colorectal cancer cell, on the peritoneum site according to the injection site. The tumour graft efficiency was compared using four grafting methods according to the PCI score, and using bioluminescence imaging in a syngeneic immunocompetent murine strain. The cells were kindly provided by the CART Laboratory (Pr Pocard Marc), INSERM U965, Paris, France.

## Material and methods

### *In vitro* cell culture

Firefly luciferase-expressing CT-26 mouse colon carcinoma cells (luc-CT26, CSC-RR0237, Creative Biogene, Shirley, NY, USA) were grown at 37°C in a 5% CO_2_ humidified atmosphere in Dulbecco’s Modified Eagle’s Medium (D6429, Dominique Dutscher, Brumath, France) supplemented with 10% foetal bovine serum (500105N1DD, Dominique Dutscher), 0.2% glucose (19002–013, Gibco, Thermo Fisher Scientific, Hampton, NH, USA), 2 mM L-glutamine (X0550, Dominique Dutscher), 100 U/ml penicillin and 100 μg/ml streptomycin (15140155, Gibco, Thermo Fisher Scientific). Cell suspensions were prepared by enzymatic treatment with trypsin–EDTA (11560626, Thermo Fisher Scientific). After centrifugation, 30 000 cells were suspended in a 200 μl injection volume of 0.9% saline solution for further orthotopic grafts.

### Development of the PM model and bioluminescence imaging

The use of animals was approved by the French National Ministry of Research (registration number, APAFIS#1 1942–2017102611003706 v2, Carcinopulse under the responsibility of S Bardet, approved by the local Ethical and Animal Care Committee, number 033). All animal care and experimental procedures were conducted in accordance with the 2013 French legislation and European Community guidelines (directive 2010/63/UE for the Care and Use of Laboratory Animals).

The BALB/cByJ strain is one of the most commonly used strains of laboratory mice. All mice were housed in ERET cages (University Limoges, France), and provided with aspen wood bedding (Lab Mix; Serlab, France), refuge mouse huts (Serlab) and cocoon products (Serlab) for environmental enrichment. Tap water and food pellets (RM1 Entretien, France) were provided *ad libitum*. The animal room was maintained under controlled temperature (21°C), photoperiod (reversed 12/12 h light/dark cycle: lights on between 19:00–07:00h) and relative humidity (50–60%) conditions, monitored by an automatic controller. All animals were checked daily, with cages changed twice per week. Female BALB/cByJ mice (8–12 weeks old, Charles River Laboratory, Paris, France) were randomly divided into four different groups (n = 10) depending on the graft injection mode: tail vein intravenous injection (IV group), left hypochondrium quadrant subcutaneous injection (SC group), intraperitoneal injection (IP group) and intraperitoneal injection after peritoneal scratching (A group). Control animals were injected with 0.9% NaCl sterile solution instead of tumour cells for each graft site.

In group A, the abdominal skin was cleaned and disinfected. The mice were chemically anesthetised by intraperitoneal injection of ketamine/xylazine solution. A midline laparotomy incision was performed using a length of approximately 1 cm. The peritoneum was gently held with forceps, and the parietal peritoneum was scraped 10 times using a sterile cotton swab [13]. The closure of the cavity was then conducted in planes, using a single running suture with 4–0 Vycril, and the skin was closed with a simple running suture with 5–0 Prolene. CT-26 cells were injected into the peritoneal cavity using a 12-gauge needle in the left hypochondrium quadrant of the abdomen.

In the IP group, the abdominal skin was cleaned and disinfected, and intraperitoneal injection of the left hypochondrium quadrant of CT-26 cells was performed using a 12-gauge needle.

In the SC group, the CT-26 tumour cell suspension was injected under the capsule of the peritoneal surface in the right upper side of the abdomen using a 12-gauge needle after cleaning and disinfecting the skin.

In the IV group, the tumour cell suspension was injected in the tail vein using a 23-gauge needle after cleaning and disinfecting the skin.

Twenty-four hours after surgery, a dose of buprenorphine was administered. All mice were checked daily for the appearance of any clinical symptoms (e.g. dyspnoea, nervousness, tonicity, hunched posture, etc.), with weight recorded every 2 days. Mice were humanely killed by CO_2_ asphyxia following the emergence of clinical symptoms, such as dyspnoea, hind limb immobility, body weight loss > 20%, or presentation of a distended abdomen (a sign of ascites).

Non-invasive bioluminescence imaging was performed on days 3, 9, 12 and 15 after luc-CT-26 grafting. First, mice under isoflurane anaesthesia were injected with 150 mg/kg XenoLight D-Luciferin (122799, PerkinElmer, Waltham, MA, USA) in 200 μl into the peritoneal cavity and, 5 min after injection, imaged with a cooled charge-coupled device camera system (IVIS Lumina System Series III, PerkinElmer). The total peak bioluminescent signal intensities in the abdominal region within regions of interest placed over the tumour on days 0 (i.e. before surgery), 3, 9, 12 and 15 post-grafting were calculated using Living Image 4.0 software (PerkinElmer). For quantitative analysis, the total signal (photons/sec) in these regions of interest was determined using Image Analyst MKII software.

### PCI after laparotomy

After 15 days, all mice were sacrificed by CO_2_ inhalation, and a complete midline laparotomy was performed immediately. The abdominal cavity was inspected and divided into 13 zones, and the PCI score was calculated as follows: 0, no macroscopic tumour; 1, limited tumour growth (1–2 mm diameter); 2, moderate tumour growth (2–4 mm diameter); and 3, abundant tumour nodules (> 4 mm diameter or > 5 deposits) [14, 15]

### Histology: Multiphoton microscopy

Tissues around the tumour site (visceral peritoneum and parietal peritoneum) were collected, fixed with 4% paraformaldehyde and embedded in paraffin. Sections (4 μm thick) were cut and stained with haematoxylin–eosin–safran and, for immunohistochemistry, with antibodies targeting CD3, CD4 and CD8. Slides were analysed using the automated BenchMark XT slide stainer (Roche, Meylan, France).

A two-photon excitation microscope was used for morphologic analysis. Anaesthetised mice and samples were positioned on the stage of the customised Olympus multiphoton microscope BX61WI/FV1200MPE (Olympus Life Science, Waltham, MA, USA) with a 25× immersion objective (1.05 NA, 2.0 mm working distance) coupled with a tuneable femtosecond Ti:sapphire pulsed laser (Chameleon Ultra II, Coherent) for excitation. Image stacks were taken at 2-μm steps and acquired under 810 nm excitation for second harmonic generation (SHG, collagen) and autofluorescence (elastin) using FluoView FV1200 software (v4.1.1.5, Olympus Life Science). The different components of the emitted light from the sample were separated using a dichroic mirror (570 nm) and detected by a pair of photomultiplier tubes preceded by fluorophore-specific emission filters (BA 575–630 for elastin, 405/10 for SHG). The obtained images were analysed using Imaris software (Bitplane AG) or Fiji/ImageJ (NIH, Bethesda, MD, USA).

### Statistical analysis

GraphPad Prism was used for all statistical analyses. The chi-square test or Fisher’s exact test was used to compare dichotomous variables, and one-way analysis of variance or the Kruskal–Wallis test was used to compare continuous variables.

## Results

All animals survived the surgical procedures without complications or infections and were monitored for 15 days after beginning the experiment. During this 2-week period, the mice did not show any physiological complications or symptoms of suffering or any apparent changes in social behaviour.

### Bioluminescence imaging and quantification of tumour growth

**Fig 1:**
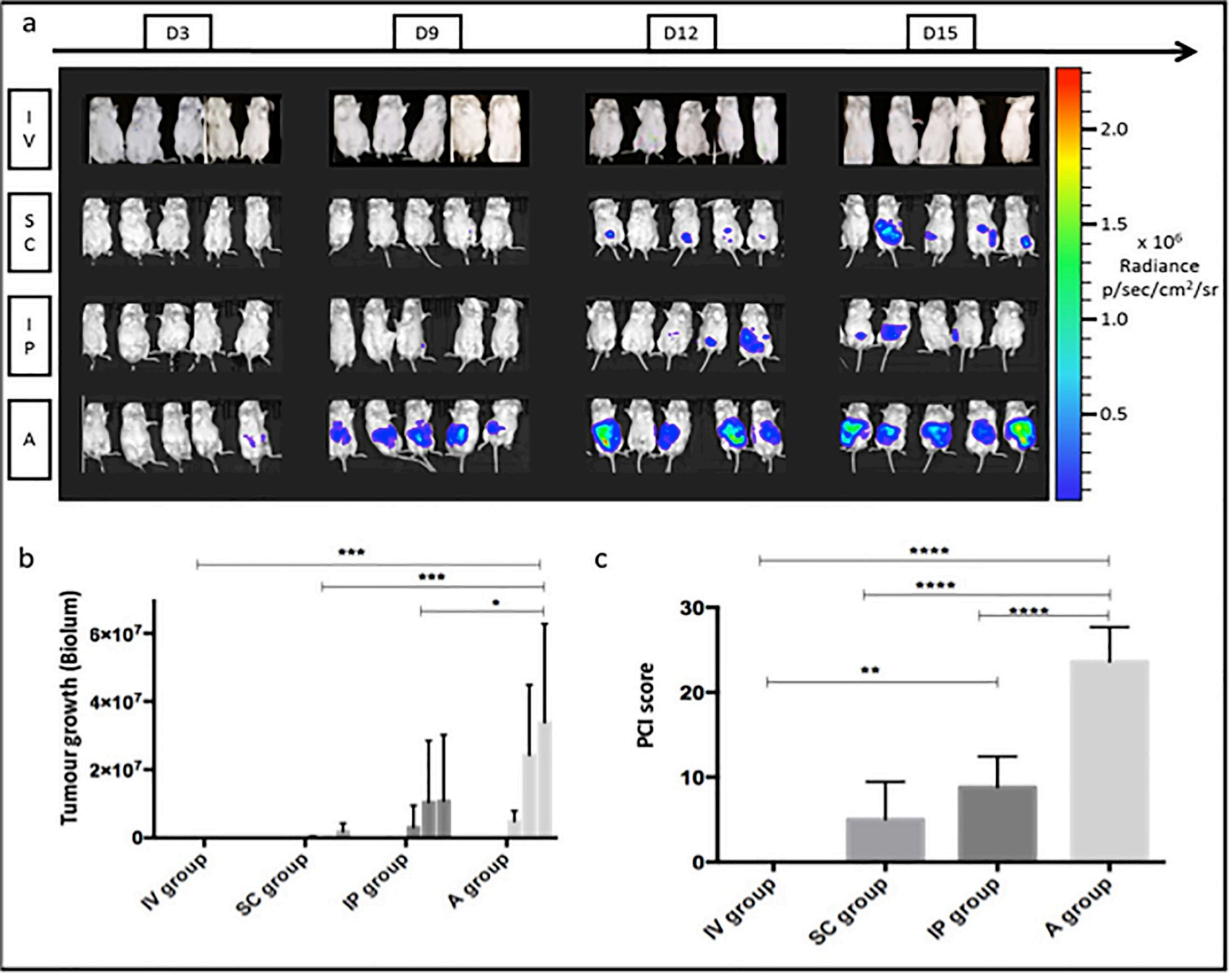
*In vivo* quantification of tumor growth by bioluminescence tracking of luciferase expressing CT-26 cells over 15 days. (A) Microphotographs of anesthetized mice for each group inside the bioluminescent imaging box. (B) Tumor growth evaluation based on bioluminescence measurements for the four groups (IV, SC, IP, A) at day 3, day 9, day 12 and day 15 (low values are not always visible on the graph). (C) PCI score at day 15 post-grafting. In summary, the mice having undergone laparotomy surgery (group A: intraperitoneal cell injection after peritoneum aggression) present an extensive tumoral growth with the highest peritoneal carcinomatosis index (PCI). Furthermore, statistically significant differences are observed between the four groups. n = 5 for each group, mean +- SD. * for p value <0.05, ** <0.01, *** <0.001 and ****<0.0001.

No mice in the IV group showed any luciferase activity by bioluminescence imaging in the abdominal cavity, as expected (Fig 1A, IV group). In the other groups, the rate of bioluminescence emission increased with time, indicating relevant tumour growth between days 3 and 15 (Fig 1A, SC, IP and A groups).

This mean bioluminescence index reflecting tumour growth was significantly higher throughout the entire experiment in the A group (mean 1.08e+7) compared with the IP group (4.049e+6, p < 0.05), SC group (341957, p < 0.001) and IV group (0, p < 0.001) (Fig 1B).

### PCI

**Fig 2:**
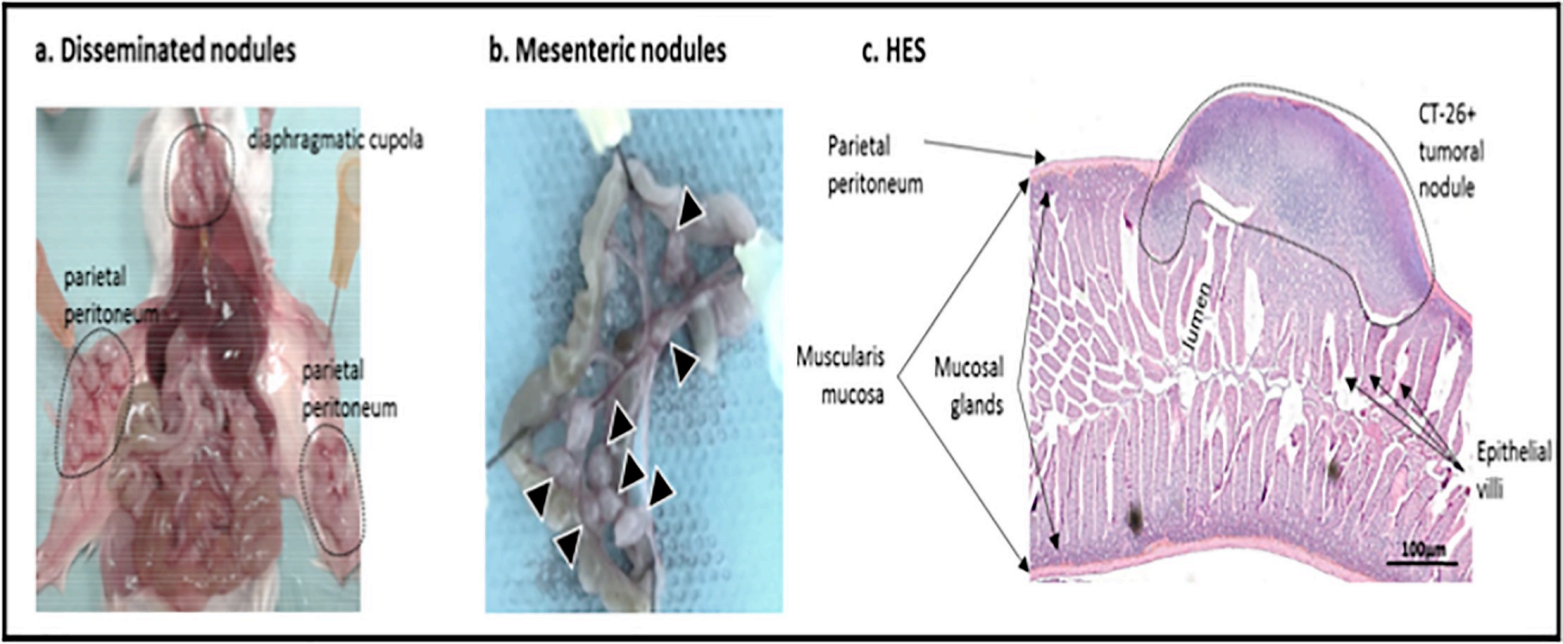
Images after mouse laparotomy at day 15 post-grafting, (A) A group showed extensive intraperitoneal tumor growth localized majority on the parietal peritoneum and the diaphragm, and (B) IP group displayed a limited peritoneal metastasis localized on the visceral peritoneum. (C) HES staining in the intestinal bowel with peritoneal tumoral nodule.

At the end of the experiment (day 15), the mean PCI score was calculated after sacrifice. The mean (± standard deviation [SD]) PCI scores were as follows: 23.6 (± 4.1), 8.8 (±3.6), 5 (± 4.5) and 0 (± 0) in the A, IP, SC and IV groups, respectively (Fig 1C, mean ± SD). The A group developed extensive intraperitoneal tumour growth localised mostly in the parietal peritoneum and diaphragm (Fig 2A). The IP group developed limited PM localised in the visceral peritoneum and mesentery (Fig 2B). The SC group developed a single nodule at the injection site, which was localised in subcutaneous tissues but not in the intra-abdominal cavity.

### Histologic analysis of normal peritoneal tissue and PM

**Fig 3:**
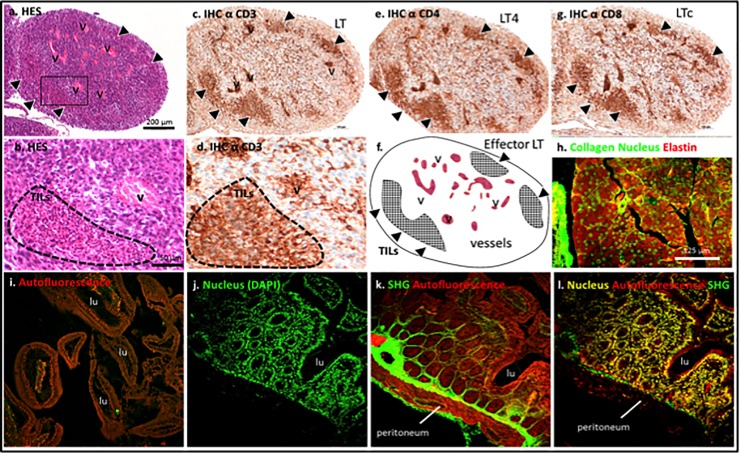
(A-B) HES staining and (C-G) immunohistochemistry for CD3, CD4, CD8 of a 15 days grafted CT-26+ nodule in a mouse from IP group. Grafted nodules reveal high number of cells including Tumor infiltrating Lymphocytes (TILs) among a complex tumoral microenvironment (high vascularization, poor fibrosis, dense tissue). Multiphoton imaging of tumoral tissue (H) and normal peritoneum (I-L). v = vessel, LT = T Lymphocyte, LT4 = T4 Lymphocyte, LTc = cytotoxic Lymphocyte, lu = lumen, SHG = second harmonic generation. Scale bar in a applied to c,e,f,g, in b applied to d, and in h applied to i-l.

Anatomic and histologic analyses corroborated the existence of various tumour nodules implanted in the intraperitoneal cavity (Figs 2C and 3A, haematoxylin–eosin–safran staining). Immunohistochemical analysis of markers specific to tumour-infiltrating lymphocytes revealed a high number of immune cells invading a complex tumour microenvironment in CT-26+ nodules on day 15 post-grafting in the IP group (Fig 3C–3G). T lymphocytes were labelled with CD3 antibodies, T4 lymphocytes with CD4 antibodies and cytotoxic T lymphocytes with CD8 antibodies. This nodule displayed high vascularisation and dense tumour tissue. In parallel, the anatomy of the tumour tissue was compared with normal peritoneal tissue by multi-photon microscopy (Fig 3H versus Fig 3I–3L, respectively). The early state of tumour development showed poor tumour fibrosis (SHG-labelling collagen) compared with the non-pathologic peritoneum.

## Discussion

Our study is the first to evaluate the experimental methods used to obtain a model of PM for exploring new therapeutic strategies. Indeed, the discovery of new therapeutics for PMc requires evaluations based on both *in vitro* and *in vivo* results that better reflect patient conditions [16, 17] First, we found that the injection site of tumour cells influences tumour growth in the peritoneum. Tumour growth was significantly greater in the A group (IP injection after peritoneal scratching). Second, this experimental study showed that the location of PMc also depends on the injection site. PMc was preferentially located in the parietal peritoneum in the A group, unlike the IP group, where it was located in the mesentery and visceral peritoneum. Third, using bioluminescence, we monitored peritoneal tumour growth and calculated luciferase activity in each animal. Luciferase activity was significantly higher in the A group compared with the other three groups.

Two principal mouse models that mimic, to some extent, the clinical situation in humans and allow direct quantitation of tumour growth and therapeutic effects have been described in the literature [18]:

i) The first is an immunocompetent syngeneic grafted mouse model, as used in our study. This model is the most commonly used to study the behaviour of PM *in vivo* (i.e. engraftment, growth and invasion) [10]. PM developed in three of our groups (IP, SC and A groups) but not after IV injection of CT-26 cells (IV group). These results confirm that IV injection of tumour cells into the tail vein produces metastasis only in the lungs [19] but is not useful for obtaining metastases at other sites, in particular, the peritoneum. Given that cancer cells are likely to spread to the liver and lung, the peritoneum could also be a destination. Most researchers agree that intraperitoneal tumours are transferred to the peritoneum by seeding [20, 21], but conclusive evidence has not been definitively established. Some human patients were found to have peritoneal carcinomatosis when the digestive tumour had not invaded the serosa, and researchers have concluded that many cancers like gynaecological cancers spread to the peritoneum through blood or the lymph vessels [22, 23]. When combined with clinical presentation, Ge et al. reported that haematogenous metastasis may be the real route of peritoneal carcinomatosis [24].

Nevertheless, we can distinguish two types of PMc according to the PCI. As might be expected, PMc may be extensive (PCI > 20), as observed in the A group. These results were confirmed by the high luciferase activity measured by bioluminescence. This method, which involves scraping out the peritoneum, was first developed in rats [25]. Our study confirmed that scratching created an environment conducive for the development of PMc in the mouse model (i.e. inflammation, growth factor release and angiogenesis for the wound-healing process) [26, 27]. This model may be used to test and validate palliative strategies, which have not yet been established, by evaluating new palliative drugs or surgical methods for extensive PMc. On the contrary, we obtained limited PMc (PCI < 10) when CT-26 cells were injected into the peritoneal cavity. Indeed, the bioluminescence levels in grafted tumours were correlated with the PCI. We chose to use an injection of 30,000 luciferase positive CT-26 cells in these experiments, as previously reported by Dico et al. (2018), given that they were able to obtain limited PMc with success at this concentration by intraperitoneal injection, after testing increasing amounts of injected cells [28]. This model is a useful uncomplicated method, most commonly used to obtain a high take rate and rapid tumour growth [10, 29]. In our experiments, this murine PMc model mimicked human PMc both macroscopically and histologically.

After SC injection, we did not observe any growth in the intra-abdominal cavity but only at the injection site above the peritoneum. These results confirm why this model is used more commonly to evaluate tumour growth but rarely produces a single large nodule with only a few or no additional PM. Nevertheless, this model has been used by many researchers for testing and analysing cytotoxic drug concentrations, penetration, and the effects, by excising this unique nodule after treatment [12, 30].

ii) The second model involves human xenografts implanted into athymic nude mice and has rarely been used in the context of PMc [30–32]. Athymic nude mice have a defective immune system because of a genetic mutation and are used in human tumour cell research [33]. Even if this model is useful for studying the physiopathology of human tumours, the interaction between the cancer and immune system cannot be observed. Our histologic results confirmed that syngeneic grafted CT-26 cells provoked an intense immune response with T cell infiltration. However, research on the immune system and anti-tumour responses has led to the development of numerous therapeutic strategies for cancer [34]. In the case of PM, the time until the tumour becomes palpable may be longer [33] and is not always compatible with animal ethics.

Bioluminescence imaging is a non-invasive imaging modality that can be employed in numerous animal studies for tracking metastasis and to measure tumour burden using luciferase-expressing tumour cells [35, 36]. We confirmed that this technique is very feasible to perform and reproducible [36, 37] Although bioluminescence has several limitations and poor sensitivity compared with positron emission tomography [38], we found a strict correlation between the PCI and bioluminescence rate. Bioluminescence imaging is an indispensable tool that can be used to investigate the dynamics of disseminated tumour growth in PM and colon cancer [39, 40], limiting the number of animals sacrificed in the study.

## Conclusions

We developed and evaluated a mouse model of PMc suitable for preclinical anti-cancerous studies. We used grafted murine colon carcinoma CT-26 cells expressing luciferase in immunocompetent BALB-c mice by IV injection (IV group), SC injection (SC group), IP injection after peritoneal scratching (A group) or IP alone (IP group). We demonstrated that limited PM could be obtained by IP injection, whereas IP injection after peritoneal scratching could lead to extensive PM.
